# Complete Response to Chemo-Immunotherapy in Recurrent Unresectable Tracheal Squamous Cell Carcinoma: A Case Report

**DOI:** 10.7759/cureus.105169

**Published:** 2026-03-13

**Authors:** Yusuke Nabe, Hiroshi Mizuuchi, Masaaki Inoue, Junichi Yoshida

**Affiliations:** 1 Department of Chest Surgery, Shimonoseki City Hospital, Shimonoseki, JPN

**Keywords:** chemoradiotherapy, immune checkpoint inhibitors, pd-l1, squamous cell carcinoma, tracheal cancer

## Abstract

Primary malignant tumors of the trachea are rare, with squamous cell carcinoma (SCC) being the most frequent subtype. Due to limited data, optimal systemic therapy for unresectable or recurrent cases remains undefined. We report a case of recurrent primary tracheal SCC achieving durable complete remission with immune checkpoint inhibitor (ICI)-based combination chemotherapy. A 73-year-old man with a history of resected lung cancer was incidentally diagnosed with tracheal SCC. Following bronchoscopic tumor debulking, he underwent concurrent chemoradiotherapy. Recurrence with pulmonary metastases occurred 4 months later. He was treated with four cycles of pembrolizumab plus nab-paclitaxel and carboplatin, followed by pembrolizumab maintenance. Complete radiologic remission was achieved and sustained for over two years. This case highlights the potential of ICI-based regimens, widely used in non-small cell lung cancer, as a promising option for recurrent tracheal SCC. Multidisciplinary approaches incorporating immunotherapy may offer meaningful benefit in this rare malignancy.

## Introduction

Primary malignant tracheal tumors are rare, accounting for approximately 2% of all upper airway tumors and only 0.1-0.4% of all cancers [1]. Of the primary tracheal cancers diagnosed, 45.9% were squamous cell carcinomas (SCC), 25.7% were adenoid cystic carcinomas, and 28.4% were other histological types. The main symptoms are dyspnea, hemoptysis, cough, and hoarseness in 55.4%, 48.6%, 41.9%, and 35.1% of patients, respectively [2]. Complete resection is widely accepted as the standard treatment for eligible patients, and the choice of surgical approach depends on factors such as tumor location, size, and whether the tumor has infiltrated the surrounding tissues [3,4]. No systematic reviews or case reports specifically focus on systemic palliative treatment for advanced tracheal malignancies. Chemotherapy regimens are generally determined based on the histological subtype, following the treatment strategy for non-small cell lung cancer [1]. Regimens used for non-small cell lung carcinoma, including concurrent chemoradiotherapy and durvalumab maintenance therapy, have also been reported for unresectable primary tracheal SCC [5]. A systematic review revealed that the survival outcomes of patients with tracheal malignancies were significantly worse in SCC than in adenoid cystic carcinoma (median overall survival: 14 months vs. 165 months; p<0.001); however, patients were not stratified by stage in the review [6]. Moreover, as tracheal cancer is a rare disease, there are few reports of immune checkpoint inhibitor (ICI)-combined chemotherapy.

Here, we report a case of tracheal cancer in which a patient relapsed after concurrent chemotherapy and survived for more than two years after receiving ICI-combined chemotherapy.

## Case presentation

**Figure 1:**
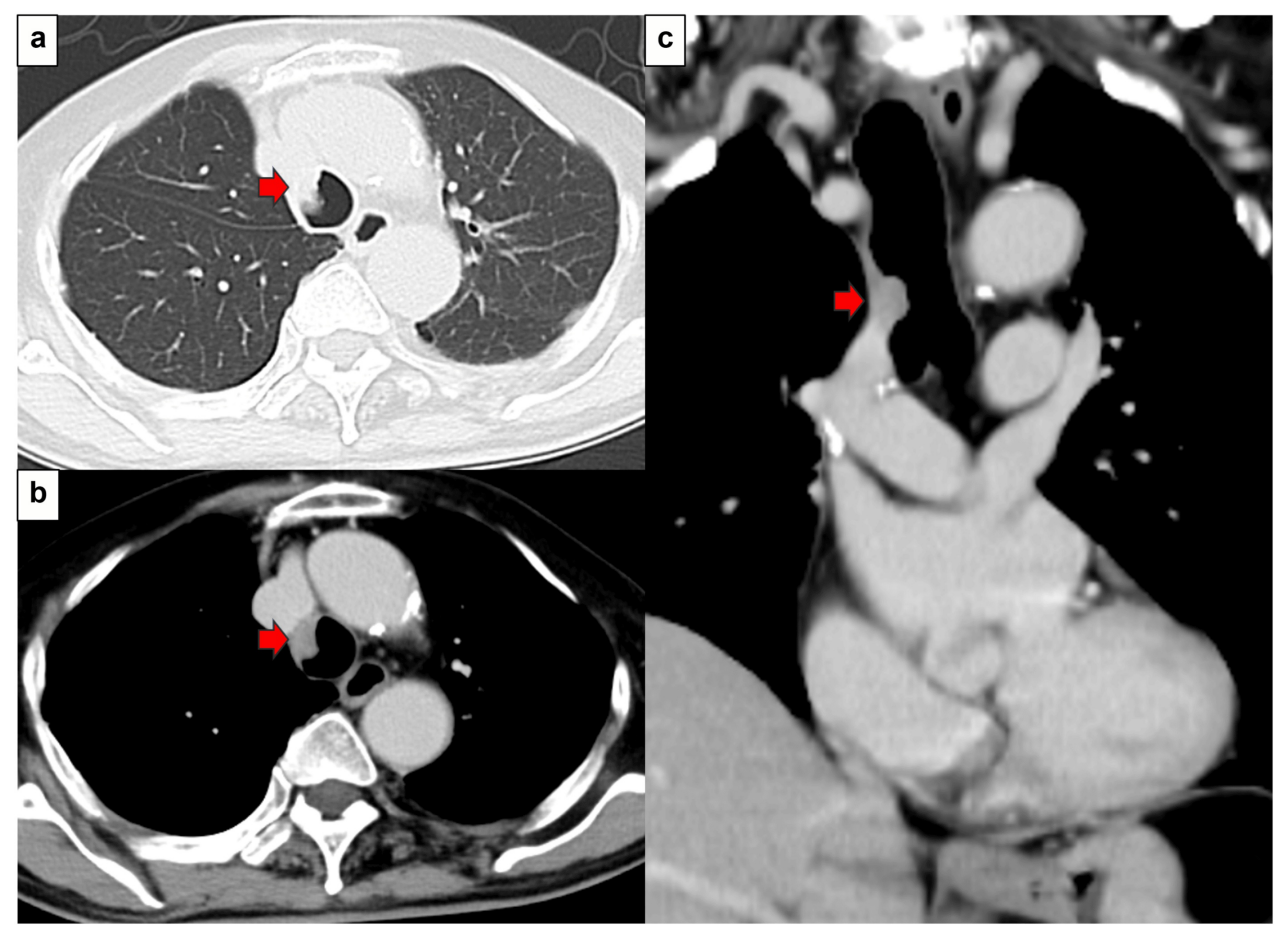
CT findings a. Lung field conditions: A nodular raised lesion was observed in the trachea. ➡：Tumor b. Mediastinal conditions. Nodular lesions were accompanied by contrast enhancement. ➡：Tumor c. Coronal section: A tumor is observed on the right side of the tracheal wall, cranial to the carina. ➡：Tumor

**Figure 2:**
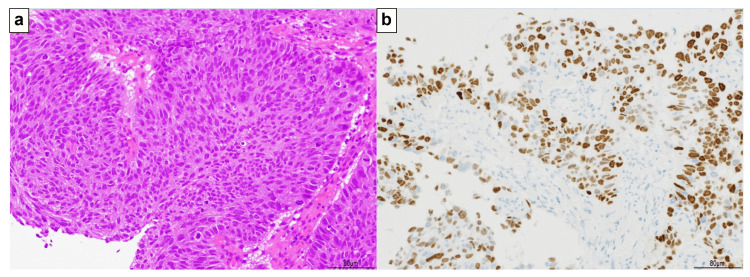
Pathological findings (at biopsy) a. Hematoxylin and eosin staining. The proliferation of large, polygonal, atypical cells showing obvious keratinization was observed. b. Immunostaining was positive for P40.

The patient was a 73-year-old male with a history of smoking (15 cigarettes/day, 40 years). The patient had no major complaints. The Eastern Cooperative Oncology Group (ECOG) performance status (PS) was 0. Two years and five months earlier, he underwent a right upper lobectomy and mediastinal lymph node dissection (ND2a-1) for right upper lobe lung cancer (pleomorphic carcinoma [containing elements of SCC; P40 positive; TTF-1, CD56, chromogranin A, and synaptophysin negative], pT2bN1M0, stage IIB). Postoperative adjuvant chemotherapy with carboplatin and Abraxane was administered for 4 weeks, and the patient has shown no evidence of lung cancer recurrence since then. Routine follow-up computed tomography (CT) revealed an endotracheal tumor (Figure 1). Hematological findings revealed negative tumor markers for SCC antigen, cytokeratin 19 fragment (CYFRA), carcinoembryonic antigen (CEA), sialylated Lex-i antigen (SLX), and pro-gastrin-releasing peptide (PRO-GRP). Bronchoscopy was performed under local anesthesia to observe the trachea. A bronchoscopic biopsy was performed, and SCC was diagnosed. Immunostaining confirmed p40 positivity and TTF-1 negativity. The tumor content was 100%, and PD-L1 (IHC 22C3) positivity was 25-49%. PD-L1 expression was assessed using the Tumor Proportion Score (TPS), defined as the percentage of viable tumor cells showing partial or complete membranous staining. A TPS of ≥1% was considered positive. The pathological findings are shown in Figure 2.

**Figure 3:**
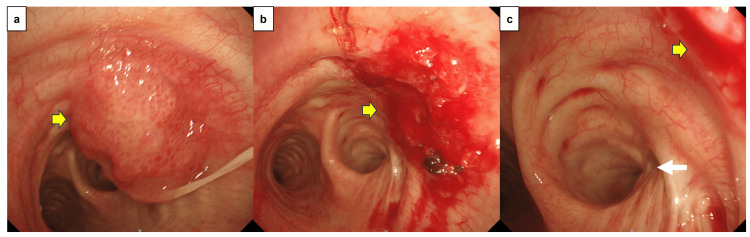
Bronchoscopic findings a. Before tumor resection, an irregular, nodular, elevated lesion accompanied by petechiae was observed in the trachea. ➡: Tumor b. After tumor removal, the tracheal stenosis was relieved, but the tumor remained in the tracheal wall. ➡: Tumor remaining in the tracheal wall c. No continuity was observed between the right upper lobe bronchial stump and the tumor. ➡: Residual tumor, ➟: Right upper lobe bronchial stump

Contrast-enhanced CT of the chest, abdomen, and pelvis, along with contrast-enhanced brain MRI, revealed no distant metastases. We diagnosed tracheal stenosis through bronchoscopic observation and decided to perform tumor resection to prevent tracheal obstruction. Bronchoscopic endotracheal tumor resection was performed under general anesthesia. Surgical findings included laser tumor resection, which relieved intratracheal stenosis. Surgery was completed with grossly incomplete resection to avoid damage to the tracheal wall (Figure 3). The specimen obtained after tumor resection was also diagnosed as SCC, consistent with the initial biopsy diagnosis.

**Figure 4:**
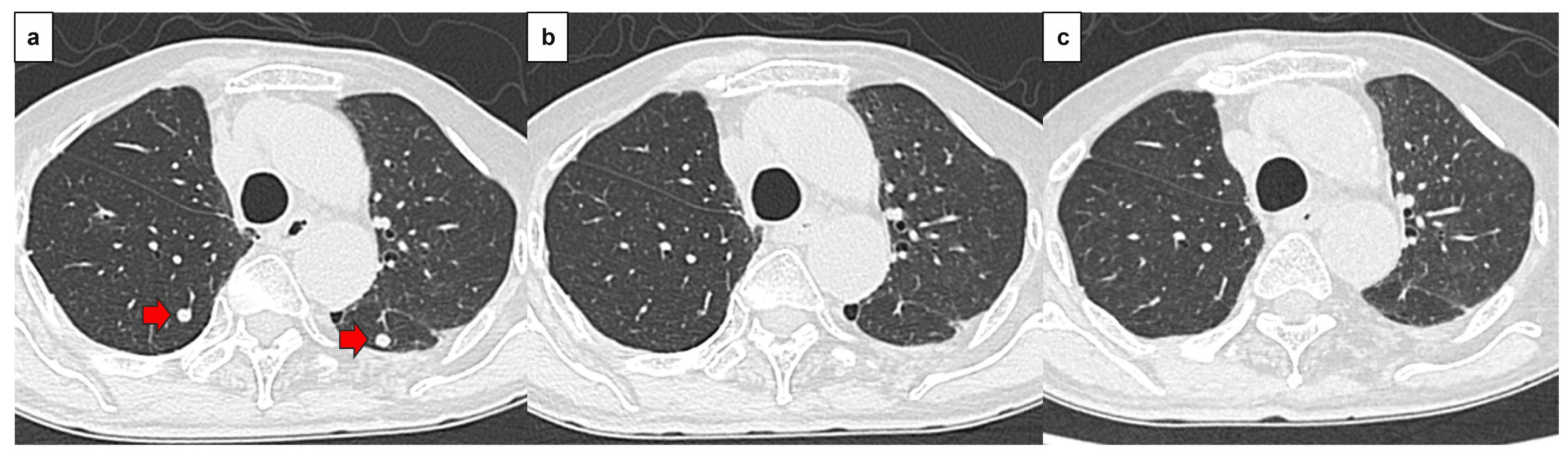
CT findings (treatment progress) a. CT findings at the time of recurrence. Multiple pulmonary nodules were observed in both lung fields. b. Two months after the start of treatment. The nodules in both lungs had shrunk and were difficult to assess. c. Two years after starting treatment. No re-enlargement of the multiple pulmonary nodules was observed.

Definitive chemoradiotherapy was administered for the residual lesions. Five weekly courses of CBDCA (AUC 2) + PTX (40 mg/m2) were administered at a total dose of 60 Gy (two Gy at a time, five times a week). Maintenance therapy with durvalumab was subsequently proposed, but the patient declined due to concerns regarding adverse events. A CT performed four months after the end of treatment revealed metastatic lung tumors, leading to a diagnosis of recurrent tracheal cancer. ECOG PS of the patient at the time of recurrence was 0. He received four courses of pembrolizumab (200 mg/body), nab-paclitaxel (100 mg/m2), and carboplatin (AUC 6), followed by maintenance therapy with pembrolizumab. Two years and nine months after the surgery, the patient remains in complete response, with no major adverse events related to treatment. CT scans of the treatment course are shown in Figure 4.

## Discussion

There are occasional reports of PET/CT scans being used for the pretreatment evaluation of tracheal cancer. However, owing to a lack of such equipment, PET/CT was not performed at our institution. Distant metastases were assessed using chest and abdominal contrast-enhanced CT and head MRI. Predicting the effectiveness of chemotherapy for tracheal cancer, a rare disease, is difficult. Bronchoscopic tumor resection was performed to treat and relieve airway stenosis. Definitive chemoradiotherapy was administered for residual disease. The patient refused to undergo maintenance ICI therapy after definitive chemoradiotherapy. There have been reports of patients with tracheal cancer receiving the PACIFIC trial [7] regimen (chemoradiotherapy followed by maintenance ICI therapy) for non-small cell lung cancer who remained disease-free for three years after treatment [5]. However, in this case, the disease recurred four months after treatment. This patient underwent surgery for right upper lobe lung cancer more than two years ago. Pathological examination revealed a diagnosis of pleomorphic carcinoma with SCC components. Differentiating primary tracheal SCC from metastatic tracheal SCC is difficult. Although endotracheal metastases from lung cancer have been reported [8], they are extremely rare. Furthermore, the recurrence of lung cancer after surgery has generally been reported within two years [9]. Bronchoscopic findings also showed no contiguous lesions in the right upper lobe of the bronchial stump, ruling out local recurrence.

Based on the clinical course described above, the patient was diagnosed with primary tracheal cancer. Although histological examination was not performed on the metastatic lung tumor at the time of recurrence, it was diagnosed as a metastatic lung tumor from tracheal cancer, as it was an early recurrence after treatment for tracheal cancer. The nodule, diagnosed as a metastatic lung tumor, disappeared during the course of chemotherapy after recurrence, and the residual lesion in the trachea did not show any signs of re-enlargement in the two years and 9 months since then. Based on the course of treatment, tracheal carcinoma and metastatic lung tumors were presumed to be clinically identical. Tracheal cancer may respond to chemotherapy for non-small cell lung cancer [5]; in this case, the risk of recurrence may have been reduced if ICI maintenance therapy had been administered after chemoradiotherapy.

This report describes a single patient, limiting generalizability, and histologic confirmation of the recurrent pulmonary lesions was not obtained, making the exact origin of metastases inferential. Larger collaborative studies and prospective evaluations are needed to clarify the role of ICI-based regimens and optimal sequencing strategies in recurrent or unresectable primary tracheal SCC.

## Conclusions

We recently reported a case of unresectable primary tracheal SCC in which a complete response was achieved with ICI-combined chemotherapy after recurrence following concurrent chemoradiotherapy. In this case, maintenance ICI therapy, which is considered effective in non-small cell lung carcinoma regimens, was not administered after concurrent chemoradiotherapy. Although the tumor recurred early, four months after treatment, a complete response was achieved with ICI-combined chemotherapy, and the patient survived without progression for two years and nine months. Multidisciplinary treatment with ICI may be effective in treating primary tracheal SCC.
